# Primary hyperparathyroidism in young patients is associated with metabolic disorders: a prospective comparative study

**DOI:** 10.1186/s12902-023-01302-9

**Published:** 2023-03-09

**Authors:** Ekaterina E. Bibik, Ekaterina A. Dobreva, Alina R. Elfimova, Anastasiia P. Miliutina, Anna K. Eremkina, Anna M. Gorbacheva, Julia A. Krupinova, Ekaterina O. Koksharova, Igor A. Sklyanik, Alexander Y. Mayorov, Natalia G. Mokrysheva

**Affiliations:** 1 Department of Parathyroid Pathology and Mineral Disorders, Endocrinology Research Centre, Dm. Ulyanova St., 11, Moscow, 117036 Russia; 2 Department of Registers of Endocrinopathies, Endocrinology Research Centre, Dm. Ulyanova St., 11, Moscow, 117036 Russia; 3 Laboratory of Clamp-Technology, Endocrinology Research Centre, Dm. Ulyanova St., 11, Moscow, 117036 Russia; 4 Endocrinology Research Centre, Dm. Ulyanova St., 11, Moscow, 117036 Russia

**Keywords:** Primary hyperparathyroidism, Calcium, Insulin resistance, Dyslipidemia, Clamp

## Abstract

**Background:**

Components of metabolic syndrome can be observed in patients with primary hyperparathyroidism (PHPT). The link between these disorders remains unclear due to the lack of relevant experimental models and the heterogeneity of examined groups. The effect of surgery on metabolic abnormalities is also controversial. We conducted a comprehensive assessment of metabolic parameters in young patients with PHPT.

**Methods:**

One-center prospective comparative study was carried out. The participants underwent a complex biochemical and hormonal examination, a hyperinsulinemic euglycemic and hyperglycemic clamps, a bioelectrical impedance analysis of the body composition before and 13 months after parathyroidectomy compared to sex-, age- and body mass index matched healthy volunteers.

**Results:**

45.8% of patients (*n* = 24) had excessive visceral fat. Insulin resistance was detected in 54.2% of cases. PHPT patients had higher serum triglycerides, lower M-value and higher C-peptide and insulin levels in both phases of insulin secretion compared to the control group (*p* < 0.05 for all parameters). There were tendencies to decreased fasting glucose (*p* = 0.031), uric acid (*p* = 0.044) and insulin levels of the second secretion phase (*p* = 0.039) after surgery, but no statistically significant changes of lipid profile and M-value as well as body composition were revealed. We obtained negative correlations between percent body fat and osteocalcin and magnesium levels in patients before surgery.

**Conclusion:**

PHPT is associated with insulin resistance that is the main risk factor of serious metabolic disorders. Surgery may potentially improve carbohydrate and purine metabolism.

## Background

The prevalence of metabolic syndrome has a tendency to increase and depends on many factors that needs the complex approach to treatment and prevention of this pathology. In the Russian Federation metabolic syndrome occurs in 40–50% of population [1]. Some epidemiological and experimental studies indicate non-classical effects of both parathyroid hormone (PTH) and calcium on adipose tissue, pancreas, vascular wall, cardiac myocytes and others, thus they may be involved in the regulation of energy processes, blood pressure and metabolism [2]. In the general population the level of PTH is positively associated with the development of metabolic syndrome and an increased risk of cardiovascular diseases. Besides, diabetes mellitus, obesity, insulin resistance (IR) and hypertension are significantly more frequent in patients with primary hyperparathyroidism (PHPT) [3, 4]. But most studies covering the problem of IR in PHPT population were based on results of indirect diagnostic methods [3, 5, 6]. The positive effect of parathyroidectomy (PTE) on metabolic parameters in patients with PHPT is inconsistent [4, 7]. This is probably due to the methodological diversity in study design. Therefore the cardiovascular and metabolic benefits of surgical treatment in PHPT population remain a subject of debate [8, 9].

Based on the above, we conducted a comprehensive assessment of metabolic parameters in young patients with PHPT using gold-standard diagnostic methods. The young age of the studied group was chosen to minimize as much as possible the impact of age-associated and other risk factors on metabolic abnormalities.

## Methods

The prospective comparative study was conducted in the Endocrinology Research Centre (Moscow, Russia) from September 2018 to October 2020. We included 24 patients with PHPT and 20 healthy sex-, age- and body mass index (BMI) matched volunteers. Inclusion criteria for patients were age ≥ 18 years, confirmed diagnosis of PHPT according to the Russian guidelines (a combination of hypercalcemia and an elevated or inappropriately normal PTH level measured twice and excluded familial hypocalciuric hypercalcemia). Exclusion criteria were following: age ≥ 50 years, severe chronic diseases (cerebrovascular disease, coronary heart disease, heart, respiratory or liver failure); decreased estimated glomerular filtration rate (eGFR) < 60 ml/min/1.73 m^2^; BMI ≥ 35 kg/m^2^; diabetes mellitus; hormone-secreting tumors of the pituitary gland, pancreas, gastrointestinal tract, adrenal glands; anamnesis of surgical operations on the pancreas; hypo-/hyperthyroidism; menstrual dysfunction for women; taking drugs affecting mineral metabolism (calcium, vitamin D metabolites, thiazide diuretics, denosumab, bisphosphonates) at the time of inclusion in the study; taking hypoglycemic therapy, somatostatin analogues, dopamine receptor agonists; mental illness; anamnesis of oncology; acute respiratory infection or exacerbation of a chronic disease during the last month; pregnancy; lactation. The control group had the same exclusion criteria and an additional one such as any parathyroid pathology.

**Fig. 1:**
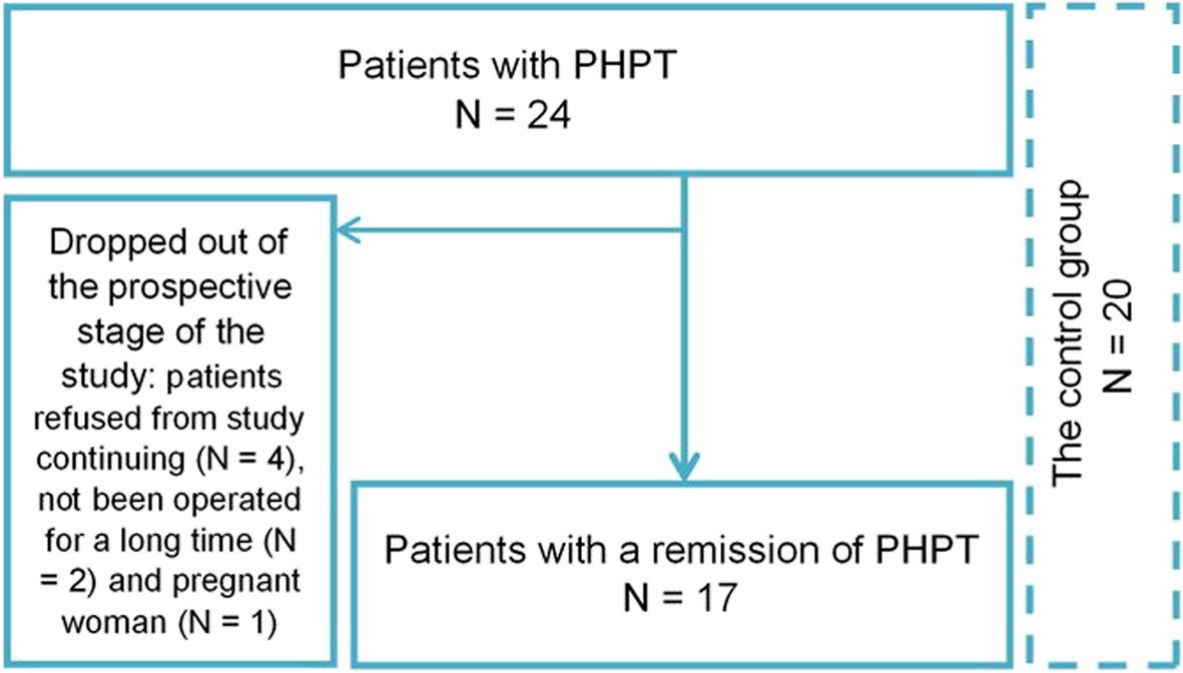
The structure of research design

Physical examination, laboratory evaluation, clamp-tests and an assessment of body composition were conducted both at baseline and 13 [10; 16] months after PTE. The study design is presented in Fig. 1.

Fasting serum biochemical parameters (total calcium (reference range (RR) 2.15–2.55 mmol/l), albumin (RR 34–48 g/l for women, 35–50 g/l for men), phosphorus (RR 0.74–1.52 mmol/l), magnesium (RR 0.7–1.05 mmol/l), creatinine (RR 50–98 µmol/l for women, 63–110 µmol/l for men), glucose (RR 3.1–6.1 mmol/l), total cholesterol (RR 3.3–5.2 mmol/l), low-density lipoprotein cholesterol (LDL, RR 1.1–3.0 mmol/l), high-density lipoprotein cholesterol (HDL, RR 1.15–2.6 mmol/l for women, RR 0.9–2.6 µmol/l for men), triglycerides (RR 0.1–1.7 mmol/l), uric acid (RR 142–339 µmol/l for women, RR 202–416 µmol/l for men), alkaline phosphatase (ALP, RR 40–150 U/l) were assessed using ARCHITECH c8000 system (Abbott, USA). Intact PTH (RR 15–65 pg/ml), C-peptide (RR 1.1–4.4 ng/ml), immunoreactive insulin (IRI, RR 2.6–24.9 µU/ml) and osteocalcin (RR 15–46 ng/ml) were measured by the electrochemiluminescence immunoassay with Cobas 6000 (Roche, Germany), serum 25 hydroxyvitamin D (25(OH)D, RR 30–100 ng/ml)—by enzyme-linked immunoassay with Liaison XL analyzer (DiaSorin, Italy). Serum leptin (µg/l) and adiponectin (µg/ml) levels were determined by enzyme-linked immunoassay using Leptin Sensitiv ELISA and Adiponectin ELISA kits (Mediagnost, Germany). Glycated hemoglobin (HbA1c, RR 4 − 6%) was measured by high performance liquid chromatography with D10 analyzer (BioRad, USA). The formula for albumin-adjusted calcium (Ca_adj_) is as follows: albumin-adjusted calcium (mmol/l) = serum calcium level (mmol/l) + 0.02 × (40 − serum albumin level (g/l)). eGFR was calculated using the CKD-EPI 2009 equation. BMI (kg/m^2^) was calculated as the ratio of weight (kg) divided by the height squared (m^2^).

All participants underwent 75 g oral glucose tolerance test (OGTT) and clamp-tests. Both clamps were performed at the morning after a 12-h overnight fast with minimum 48-h interval between them. IR was measured by the classic DeFronzo hyperinsulinemic euglycemic clamp-test. It included continuous IV infusion of regular insulin at a constant rate of 1 mIU/kg/min using a syringe pump (Perfusor compact, B. Braun, Germany). Simultaneously, 20% glucose solution was infused using Infusomat fmS (B. Braun, Germany) to maintain blood glucose level in the range 5.1–5.6 mmol/l, that was controlled every 5 min with a glucose analyzer (OneTouch “VerioPro + ”, Switzerland). Glucose uptake by tissues (M-value) was calculated as an average amount of glucose (mg/kg/min) required to maintain glycemic targets during 30 min of dynamic equilibrium of the glucose infusion rate. M-values were classified into 4 groups: 0–2 (severe IR), 2–4 (moderate IR), 4–6 (mild IR), and > 6 (no IR). Two-phase insulin secretion was measured using a modification of DeFronzo hyperglycemic clamp test. 20% glucose IV infusion consisted of two stage: 1) a 15-min “priming dose” to raise blood glucose level to hyperglycemic plateau; 2) a “maintenance dose”. The “priming dose” was calculated per body surface area (m^2^) and equal to 9,622 mg/m^2^. Then glucose solution was infused to maintain blood glucose level in the range 9.3–10.7 mmol/l during 2 h under the control every 5 min with a glucose analyzer. Every 2 min of the first stage and every 10 min of the second stage blood samples were obtained to evaluate serum C-peptide and IRI levels. The first phase of insulin secretion was assessed by areas under curves (AUC) of C-peptide and IRI concentrations during the priming stage and the second one by respective AUC during the last test period. Multi-frequency bioelectrical impedance analysis of body composition was performed using InBody-770 analyzer (Inbody Co., LTD, North Korea).

Statistical analysis was performed using Statistica 13.0 (StatSoft, USA) and SPSS (IBM, USA) software packages. Descriptive statistics of quantitative characteristics are presented by medians and interquartile ranges (Median, IQR [25;75]%), descriptive statistics of qualitative characteristics—in absolute and relative frequencies. The Mann–Whitney test (U-test) and Chi-square test were applied to compare two independent groups in terms of quantitative and qualitative characteristics respectively. The Wilcoxon signed-rank test (W-test) was used to compare related groups. Spearman’s rank correlation coefficient was applied for the examination of variable correlations. The critical level of significance when testing statistical hypotheses was equal to 0.05.

## Results

The ratio of men and women in the PHPT group before surgery was 1:4 with the median age 37 [33; 41] years and median illness duration (since the first symptoms or laboratory test abnormalities) 3 [1.5; 7] years. The baseline laboratory parameters of mineral metabolism of both groups are presented in Table 1.

**Table 1:** The laboratory parameters of mineral metabolism of both groups at baseline

Parameters	PHPT group (*n* = 24)	Control group (*n* = 20)	p, U-test
PTH, pg/ml	141 [111; 228]	39.9 [33.8; 47.5]	< 0.001
Ca_adj_, mmol/l	2.73 [2.61; 2.94]	2.23 [2.15; 2.28]	< 0.001
Phosphorus, mmol/l	0.76 [0.73; 0.84]	1.14 [1.09; 1.25]	< 0.001
Magnesium, mmol/l	0.84 [0.79; 0.86]	0.81 [0.78; 0.82]	0.014
eGFR in CKD-EPI, ml/min/1.73 m^2^	104 [94; 111]	102 [95; 106]	0.579
ALP, U/l	71 [60; 85]	54 [41; 61.5]	< 0.001
Osteocalcin, ng/ml	46.0 [38.2; 67.7]	19.3 [16.6; 21.5]	< 0.001
25(OH)D, ng/ml	19.0 [13.3; 21.9]	20.8 [17.1; 28.2]	0.154

Data are presented by medians and interquartile ranges (Median, IQR [25;75]%). U-test: the Mann–Whitney test;

*PHPT* Primary hyperparathyroidism, *PTH* Parathyroid hormone, *Ca*_*adj*_ Albumin-adjusted calcium, *eGFR* Estimated glomerular filtration rate, *ALP* Alkaline phosphatase, *25(OH)D* 25 Hydroxyvitamin D

*p* < 0.05 was considered statistically significant

According to the OGTT results, only 1 patient had an impaired glucose tolerance with normal HbA1c, others did not show any carbohydrate metabolism abnormalities. Various types of dyslipidemia and hyperuricemia were detected in 54.2% (*n* = 13) and in 29.2% (*n* = 7) of PHPT patients respectively. 11 patients were overweight (45.8%) and the one was obese (BMI 31.8 kg/m^2^). 45.8% of patients had excessive visceral fat (the area of ​​visceral adipose tissue > 100 cm^2^), including those with normal BMI. IR was diagnosed in 54.2% of patients (*n* = 13), four of them presented with moderate IR, none had severe IR. The baseline metabolic parameters of both groups are presented in Table 2.

**Table 2:** The baseline metabolic parameters of both groups

Parameters	PHPT group (*n* = 24)	Control group (*n* = 20)	p, U-test
BMI, kg/m^2^	24.6 [22.5; 26.5]	23.9 [22.7; 25.9]	0.612
Fasting glucose, mmol/l	5.04 [4.63; 5.23]	4.83 [4.50; 5.20]	0.556
2-h glucose OGTT, mmol/l	5.51 [4.78; 6.34]	4.89 [4.40; 5.74]	0.260
HbA1c, %	5.30 [5.10; 5.50]	5.20 [5.10; 5.50]	0.815
Total cholesterol, mmol/l	4.94 [4.49; 5.41]	5.18 [4.36; 5.46]	0.732
LDL cholesterol, mmol/l	3.04 [2.58; 3.69]	3.39 [2.36; 3.62]	0.962
HDL cholesterol, mmol/l	1.35 [1.14; 1.75]	1.53 [1.26; 1.76]	0.494
Triglycerides, mmol/l	1.13 [0.94; 1.39]	0.79 [0.63; 1.01]	0.001
Uric acid, µmol/l	298 [246; 366]	253 [231; 311]	0.134
Adiponectin, µg/ml	7.22 [4.56; 8.79]	7.23 [4.81; 10.8]	0.849
Leptin, µg/l	12.5 [4.74; 18.8]	7.09 [6.28; 11.7]	0.247
M-value, mg/kg/min	5.60 [4.25; 7.45]	7.90 [7.00; 10.6]	0.001
AUC C-peptide phase 1	61.9 [44.4; 73.9]	37.6 [36.1; 42.6]	< 0.001
AUC C-peptide phase 2	160 [145; 198]	132 [115; 175]	0.019
AUC IRI phase 1	648 [438; 834]	294 [259; 384]	< 0.001
AUC IRI phase 2	1150 [961; 1448]	760 [657; 1012]	0.001
Percent body fat, %	31.5 [20.9; 36.2]	31.2 [25.6; 33.7]	0.916
Visceral fat area, cm^2^	89.9 [61.3; 120]	88.1 [75.9; 106]	0.770

Data are presented by medians and interquartile ranges (Median, IQR [25;75]%). U-test: the Mann–Whitney test

*PHPT* Primary hyperparathyroidism, *BMI* Body mass index, *OGTT* Oral glucose tolerance test, *HbA1c* Glycated hemoglobin, *LDL* Low-density lipoprotein, *HDL* High-density lipoprotein, *AUC* Area under curve, *IRI* Immunoreactive insulin

*p* < 0.05 was considered statistically significant

**Fig. 2:**
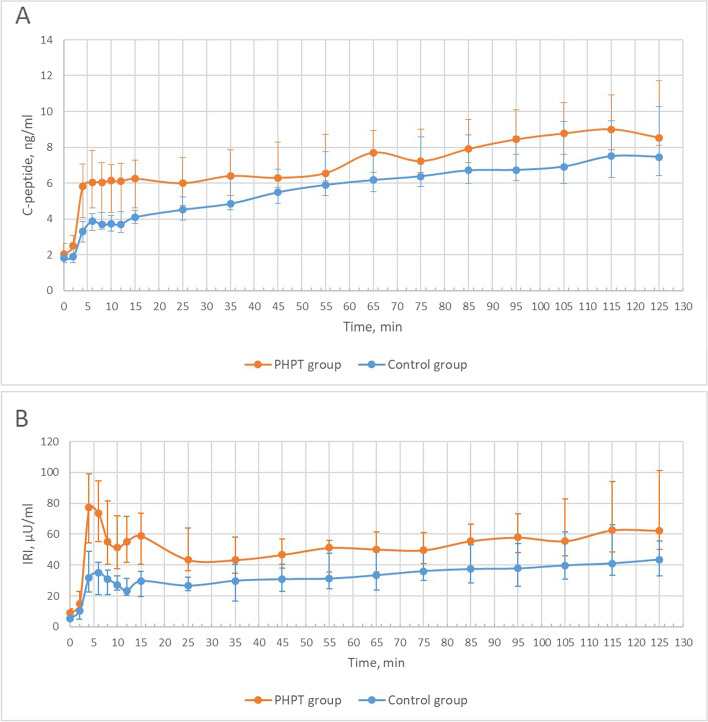
Changes of parameters during the hyperglycemic clamp at baseline in examined groups

Patients with PHPT had higher serum triglycerides levels without any differences in other metabolic parameters compared to the control group. They also showed lower M-value, higher serum C-peptide and IRI concentrations in both phases of insulin secretion. Changes of the parameters during the hyperglycemic clamp at baseline are presented in Fig. 2A, B.

**Fig. 3:**
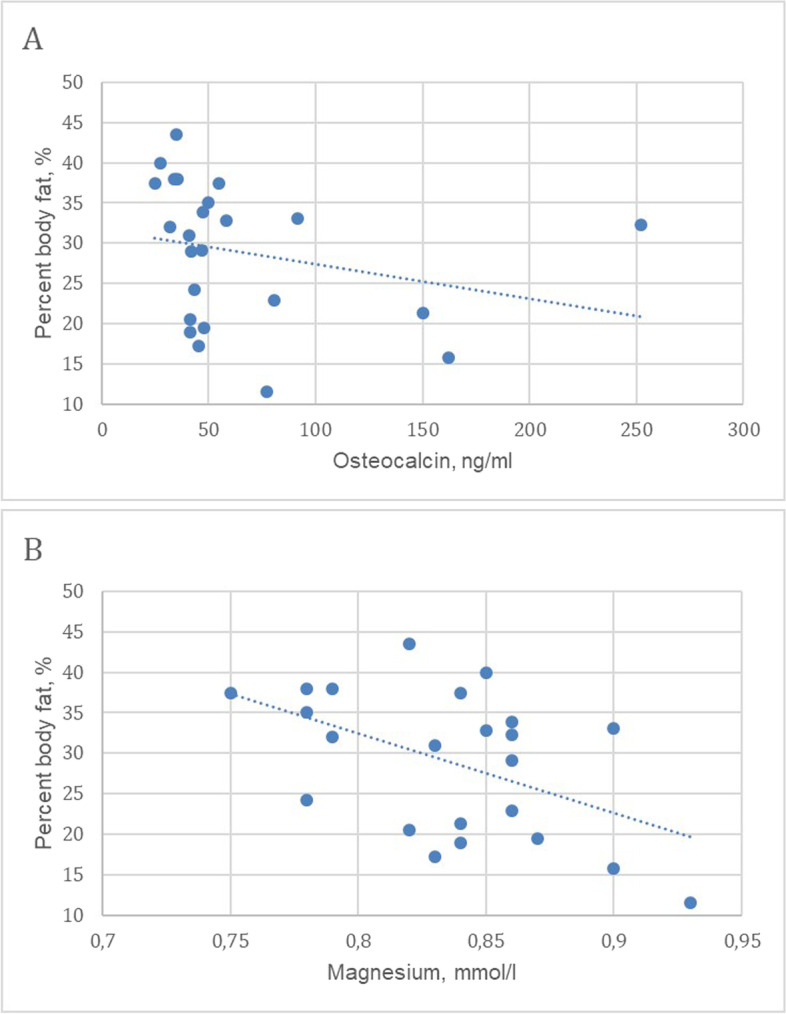
Correlations of percent body fat with parameters of mineral metabolism in patients with PHPT at baseline

**Fig. 4:**
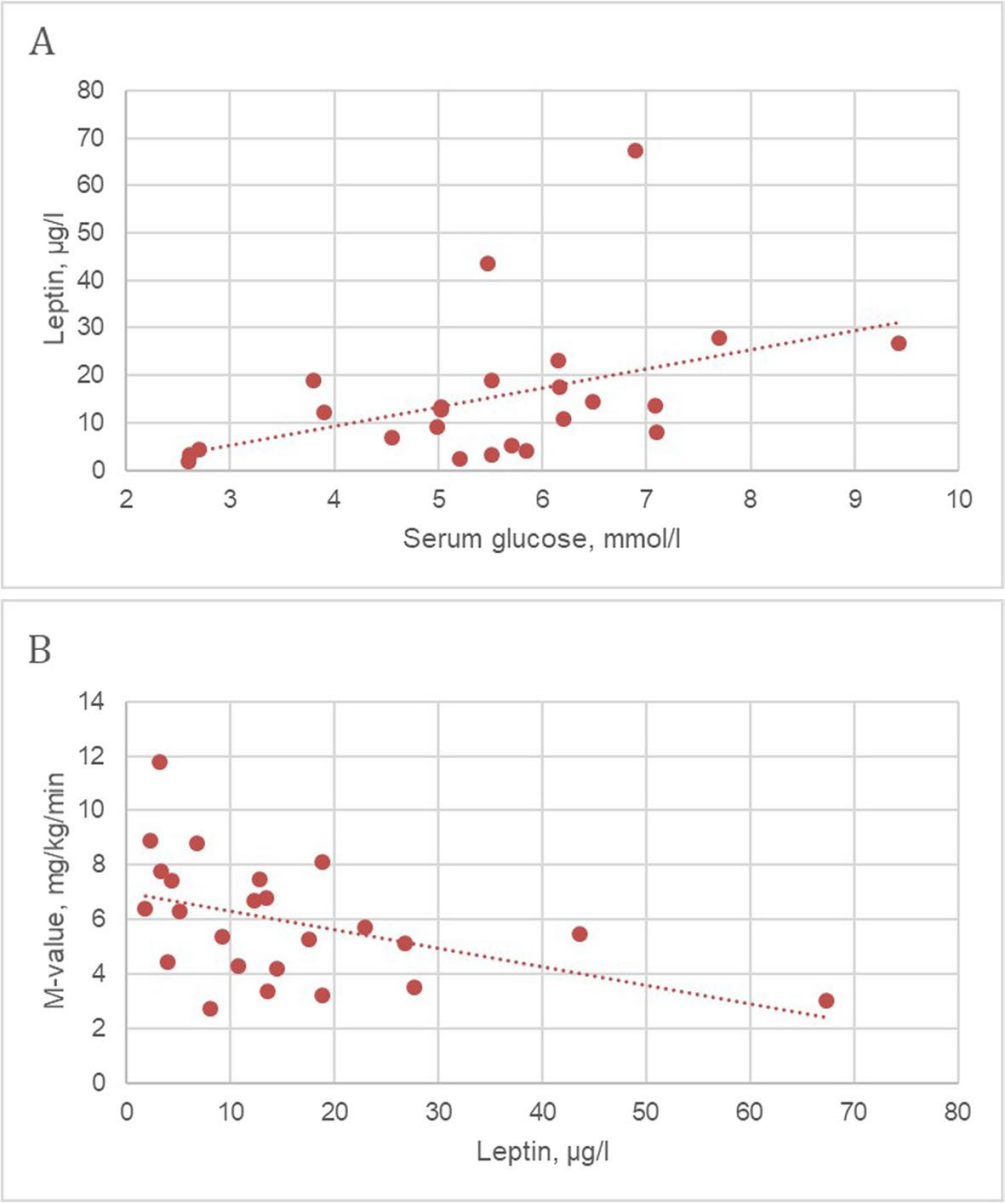
Correlations of serum leptin level with parameters of carbohydrate metabolism in patients with PHPT at baseline

We found a negative correlation between percent body fat (PBF) and osteocalcin and magnesium levels (Fig. 3A, B) in the PHPT group. 2-h OGTT glucose level had direct links with PBF and ​​ visceral fat area (VFA) (*r*_1_ = 0.42, *p*_1_ = 0.040 and *r*_2_ = 0.44, *p*_2_ = 0.031, respectively), M-value had the opposite links with these body composition parameters (*r*_1_ = -0.49, *p*_1_ = 0.014 and *r*_2_ = -0.42, *p*_2_ = 0.041, respectively). Concentrations of C-peptide and IRI in both secretion phases demonstrated apparent correlations with BMI. Serum leptin level had a positive correlation with 2-h OGTT glycaemia and a negative with M-value (Fig. 4A, B).

Only 17 patients continued the study after radical PTE. The re-examination was completed within 13 [10; 16] months, min 7 months, max 19 months. The follow-up data of mineral and metabolic parameters are presented in Tables 3 and 4. In the post-surgery period we detected decreased fasting glucose, uric acid levels and IRI concentration of the second secretion phase but no significant changes of lipid profile and M-value. BMI and HbA1c had been increased but body composition of patients did not change significantly.

**Table 3:** The follow-up laboratory parameters of mineral metabolism in the PHPT group

Parameters	PHPT group (*n* = 17)		p, W-test
	Baseline	After surgery	
PTH, pg/ml	138 [106; 210]	38.8 [32.7; 49.2]	< 0.001
Ca_adj_, mmol/l	2.71 [2.61; 2.91]	2.18 [2.16; 2.24]	< 0.001
Phosphorus, mmol/l	0.76 [0.72; 0.83]	0.94 [0.87; 1.07]	0.001
Magnesium, mmol/l	0.84 [0.82; 0.86]	0.80 [0.77; 0.84]	0.031
eGFR in CKD-EPI, ml/min/1.73 m^2^	102 [95; 109]	100 [91; 108]	0.070
ALP, U/l	76.0 [65.0; 108]	42.0 [36.0; 56.0]	0.001
Osteocalcin, ng/ml	46.7 [41.0; 54.6]	17.6 [11.4; 23.2]	< 0.001
25(OH)D, ng/ml	20.5 [16.6; 22.2]	30.4 [22.2; 36.5]	0.004

Data are presented by medians and interquartile ranges (Median, IQR [25;75]%). W-test: the Wilcoxon signed-rank

*PHPT* Primary hyperparathyroidism, *PTH* Parathyroid hormone, *Ca*_*adj*_ Albumin-adjusted calcium, *eGFR* Estimated glomerular filtration rate, *ALP* Alkaline phosphatase, *25(OH)D* 25 Hydroxyvitamin D

*p* < 0.05 was considered statistically significant

**Table 4:** The follow-up metabolic parameters of the PHPT group

Parameters	PHPT group (*n* = 17)		p, W-test
	Baseline	After surgery	
BMI, kg/m^2^	25.0 [23.3; 26.8]	25.3 [23.8; 27.5]	0.004
Fasting glucose, mmol/l	5.10 [4.81; 5.24]	4.69 [4.48; 5.00]	0.031
2-h glucose OGTT, mmol/l	5.51 [4.56; 6.48]	5.48 [4.74; 7.22]	0.379
HbA1c, %	5.30 [5.10; 5.50]	5.60 [5.30; 5.80]	0.001
Total cholesterol, mmol/l	4.89 [4.19; 5.59]	5.08 [4.5; 5.61]	0.246
LDL cholesterol, mmol/l	2.90 [2.50; 3.64]	3.23 [2.4; 3.8]	0.381
HDL cholesterol, mmol/l	1.34 [1.14; 1.76]	1.33 [1.15; 1.67]	0.795
Triglycerides, mmol/l	1.16 [1.02; 1.35]	1.22 [0.93; 1.63]	0.278
Uric acid, µmol/l	307 [254; 357]	260 [238; 352]	0.044
Adiponectin, µg/ml	8.08 [6.27; 9.71]	7.10 [3.98; 10.3]	0.059
Leptin, µg/l	10.8 [4.36; 17.6]	12.1 [4.09; 24.8]	0.123
M-value, mg/kg/min	5.48 [4.30; 7.43]	6.17 [4.56; 6.90]	0.959
AUC C-peptide phase 1	63.2 [47.5; 73.5]	53.9 [44.2; 71.0]	0.679
AUC C-peptide phase 2	161 [149; 193]	169 [139; 196]	0.737
AUC IRI phase 1	657 [426; 862]	501 [339; 768]	0.163
AUC IRI phase 2	1121 [917; 1320]	982 [806; 1375]	0.044
Percent body fat, %	37.6 [25.8; 41.7]	31.7 [22.5; 37.1]	0.266
Visceral fat area, cm^2^	88.1 [56.6; 140]	87.8 [59.7; 141]	0.149

Data are presented by medians and interquartile ranges (Median, IQR [25;75]%). W-test: the Wilcoxon signed-rank

*PHPT* Primary hyperparathyroidism, *BMI* Body mass index, *OGTT* Oral glucose tolerance test, *HbA1c* Glycated hemoglobin, *LDL* Low-density lipoprotein, *HDL* High-density lipoprotein, *AUC* Area under curve, *IRI* Immunoreactive insulin

*p* < 0.05 was considered statistically significant

**Fig. 5:**
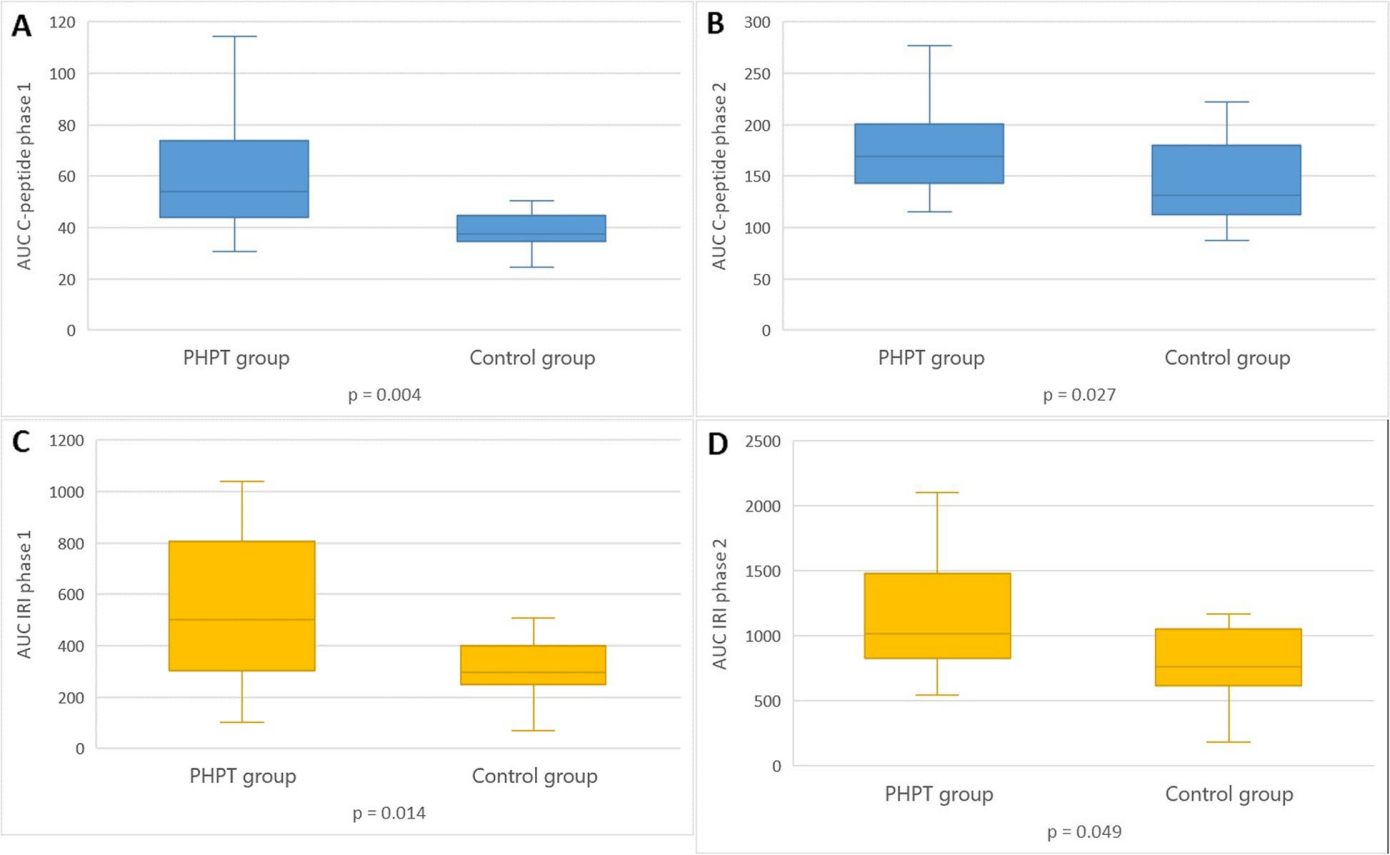
The comparisons of serum C-peptide and IRI concentrations in patients with PHPT after PTE and the control group. U-test: the Mann-Whitney test; p< 0.05 was considered statistically significant. *AUC* Area under curve, *PHPT* Primary hyperparathyroidism, *IRI* Immunoreactive insulin

The PHPT group after PTE still had higher serum triglycerides (*p* = 0.002) and HbA1c (*p* = 0.011) compared to the control group. Also lower M-value (*p* = 0.001) and higher serum C-peptide and IRI concentrations in both phases of pancreas secretion persisted in patients with previous PHPT compared to the healthy volunteers (Fig. 5A-D).

## Discussion

The main aim of our study was to estimate the main metabolic parameters in the young PHPT population using the most specific diagnostic methods. We revealed IR in more than half of young patients with PHPT as well as lower M-value and higher serum IRI concentrations compared to people without parathyroid pathology. This fact may be explained by the differences in mineral metabolism between groups because body composition was comparable. Majority of colleagues confirmed a reduction in insulin sensitivity accompanied by increased insulin secretion in PHPT using indirect estimated indices (HOMA-IR, HOMA-B, QUICKI, ISI). It is assumed that serum calcium level contributes to impaired insulin sensitivity [3, 5, 6]. Some researchers demonstrated the suppressed effect of PTH on glucose uptake by tissues [10, 11]. Cvijovic G. et al. used an euglycemic clamp in patients with PHPT too but did not detect difference in M-value and parameters of insulin secretion compared to healthy controls [12]. Using a hyperglycemic clamp technique Prager R. et al. found that insulin secretion was significantly elevated in patients with PHPT compared to controls [13]. Unfortunately, we did not find any significant correlations of PTH or other mineral markers and M-value, IRI or C-peptide levels but it could be because of the small sample size.

We also revealed dyslipidemia in more than half of PHPT patients and especially higher serum triglycerides compared to the control group. Hyperparathyroidism may impair the lipid elimination by decrease in lipoprotein lipase activity [14]. 

There were no correlations between carbohydrate metabolism parameters and serum calcium or PTH. Probably there is nonobvious link between them because follow-up in the post-surgical period showed a decrease in fasting glucose.

There was a negative correlation between osteocalcin level and PBF in our patients before surgery (accompanied with VFA). This is similar to Chinese study that showed an inverse relationship between serum osteocalcin levels and VFA [15]. Gianotti L. et al. demonstrated osteocalcin was negatively associated with fasting glucose and positively associated with HOMA2-S% before PTE. But after PTE osteocalcin levels had significantly decreased, while HOMA2-S% did not change [16]. Mendonça M. L. et al. did not observe associations between osteocalcin and insulin, glucose levels, HOMA-IR in PHPT group [17]. Therefore, the role of bone remodeling in metabolic processes requires further investigation.

Despite the previously described changes of leptin and adiponectin concentrations in PHPT [18–20], we did not confirm them as well as a statistically significant increase in uric acid compared to the healthy volunteers [21]. Probably this is because of restricted sample size.

The studies demonstrated controversial effects of PTE on metabolic parameters in PHPT [22–27]. Our results are more consistent with data of the recent systematic review. PTE could improve glycemic parameters (including insulin concentration) despite increased BMI but not lipid exchange [28]. We also confirmed the results of Prager R. et al., who did not observe the difference in M-value but noted a decrease in insulin secretion in patients with PHPT before and after surgery [13] whereas Cvijovic G. et al. got opposite results [12]. The differences are more likely determined by various observation periods. We revealed the decrease of uric acid level after surgery similar to results of the meta-analysis [21].

Limitations of the study.

The limitations of our study are the small sample sizes. Besides, we included 2 patients with *MEN1* mutation that itself may affect carbohydrate balance. Wijk J.P.H. et al. supposed decreased IR and higher prevalence of impaired fasting glucose were unrelated to MEN-1 manifestations [29]. Another limitation is conducting research during COVID-19 pandemic. According to some studies, COVID-19 is associated with increased blood glucose including people without pre-existing diabetes [30, 31]. The tendency to hyperglycemia because of transient damage of pancreatic islets can persist after recovery from the disease [31, 32]. In addition, COVID-19 pandemic affected the time of examination of patients after surgery, which could also impact the results of the study.

## Conclusion

Changes of bone and mineral parameters can lead to metabolic disorders in young patients with PHPT. PHPT is associated with IR, which is the main risk factor for the severe carbohydrate and fat disorders. The tendency to hypertriglyceridemia in patients with parathyroid tumors compared to the healthy volunteers suggests the contribution of disease to dyslipidemia. Remission of PHPT could improve the carbohydrate and purine balance but further studies are required to clarify this fact.

## Data Availability

All data generated or analyzed during this study are included in this published article.

## Abbreviations

ALP: Alkaline phosphatase
AUC: Area under curve
BMI: Body mass index
Ca_adj_: Albumin-adjusted calcium
eGFR: Estimated glomerular filtration rate
HbA1c: Glycated hemoglobin
HDL: High-density lipoprotein (cholesterol)
IQR: Interquartile ranges
IR: Insulin resistance
LDL: Low-density lipoprotein (cholesterol)
OGTT: Oral glucose tolerance test
PBF: Percent body fat
PHPT: Primary hyperparathyroidism
PTE: Parathyroidectomy
PTH: Parathyroid hormone
RR: Reference range
VFA: Visceral fat area
25(OH)D: 25 Hydroxyvitamin D
